# Self-Harm by Nurses and Midwives – A Study of Hospital Presentations

**DOI:** 10.1027/0227-5910/a000936

**Published:** 2024-01-18

**Authors:** Samantha Groves, Karen Lascelles, Liz Bale, Fiona Brand, Deborah Casey, Keith Hawton

**Affiliations:** ^1^ Centre for Suicide Research, Department of Psychiatry, University of Oxford, Warneford Hospital, Oxford, UK; ^2^ Mental Health Directorate, Oxford Health NHS Foundation Trust, Warneford Hospital, Oxford, UK

**Keywords:** nurses, self-harm, methods, problems, aftercare

## Abstract

**Abstract:** *Background*: Nursing professionals are an occupational group at increased risk of suicide, but little is known about self-harm in this population. *Aims*: To investigate the characteristics of nurses and midwives who present to hospital following self-harm. *Method:* We used data from the Oxford Monitoring System for Self-Harm to identify nurses and midwives who presented to the general hospital in Oxford during 2010–2020 following an episode of self-harm and received a psychosocial assessment. *Results:* During the eleven-year study period, 107 presentations of self-harm involving 81 nurses and midwives were identified. Self-poisoning was the most common self-harm method (71.6%), with antidepressants and paracetamol most frequently involved. Many had consumed alcohol before (43.8%) or during (25.3%) the self-harm act. Some individuals had high or very high suicide intent scores (22/70, 31.4%). Common problems preceding self-harm included problems with a partner (46.9%), psychiatric disorder (29.6%), and problems with employment (27.2%), family (24.7%), and alcohol (23.5%). A range of aftercare options were offered following presentation. *Limitations:* This study was limited to data from a single hospital. *Conclusion:* Prevention and management of self-harm within this occupational group requires preventative strategies and availability of interventions addressing the range of factors that may contribute to self-harm, especially relationship problems, psychiatric disorders, employment problems, and alcohol misuse.

Research in several countries, including the United Kingdom and the United States, has shown that nurses, particularly female nurses, are at increased risk of suicide compared to the general population or other occupational groups (e.g., Davidson et al., 2020; Windsor-Shellard & Gunnell, 2019). In England, 1.6% of suicides between 2011 and 2016 involved nurses or midwives (The National Confidential Inquiry into Suicide and Safety in Mental Health [NCISH], 2020).

Nonfatal self-harm is strongly linked to suicide risk (Carroll et al., 2014), especially shortly after hospital-presenting self-harm (Geulayov et al., 2019). Prior self-harm is common among nurses who die by suicide in the United Kingdom (Hawton et al., 2002; NCISH, 2020). However, there has been little research on self-harm in nurses. Of a sample of nurses in Hong Kong, 9.3% reported self-harm in the previous year, most commonly by cutting, hitting, or poisoning (Cheung & Yip, 2016). Factors associated with self-harm were having less than 10 years of clinical experience, chronic illness, relationship crises, family history of self-harm, smoking, psychiatric disorder, and stress (Cheung & Yip, 2016). To the authors’ knowledge, self-harm among nurses in the United Kingdom has not yet been examined.

We have utilized a well-established general hospital self-harm monitoring system to identify and study a series of nurses and midwives who presented to hospital following self-harm between 2010 and 2020. The aim was to investigate the characteristics of nurses and midwives who self-harm, including their demographic characteristics, methods of self-harm, problems preceding self-harm, and aftercare offered.

## Methods

### Study Sample

The study population included current or former nurses and midwives who presented to the general hospital in Oxford between January 1, 2010, and December 31, 2020, following an episode of self-harm and received a psychosocial assessment. Nursing and midwifery students were not included due to inconsistency in reporting of students’ programs of study within medical records.

### Data Collection

Data were collected using the long-established Oxford Monitoring System for Self-harm (e.g., Hawton et al., 2015). Self-harm includes nonfatal intentional self-poisoning, self-injury, or a combination of these methods, irrespective of whether there is evidence that the act was intended to result in death (Hawton et al., 2003). Self-poisoning is defined as the intentional self-administration of more than the prescribed dose of any drug. It also includes poisoning with noningestible substances and gas. Overdoses of recreational drugs and severe alcohol intoxication are included where the clinical staff consider these are cases of self-harm. Self-injury is defined as any injury that has been deliberately self-inflicted.

Patients who present at the emergency department in the general hospital in Oxford following an episode of self-harm are usually assessed by a specialist mental health clinician and data are gathered concerning their sociodemographic (e.g., sex, age) and clinical characteristics (e.g., psychiatric history, alcohol misuse), self-reported life problems (e.g., interpersonal problems), together with a record of what aftercare is offered. Previous self-harm data are based on hospital and nonhospital presenting self-harm episodes. Information is gathered from reports by the patient during their index psychosocial assessment, patient medical records, and data from the self-harm monitoring system. During the assessment, staff usually also complete the Beck Suicide Intent Scale, a 15-item measure examining the severity of suicide intention in relation to self-harm (Beck et al., 1974; Harriss et al., 2005). The scale comprises two parts: The first, involving eight items, assesses the objective *circumstances* of the self-harm act, and the second is a seven-item *self-report* assessment of the patient’s thoughts and feelings at the time of self-harm. Each item has a score range of 0–2, with a maximum score of 30. For this study, intent was classified as low (score 0–6), moderate (7–12), high (13–20), and very high (21+; Hawton, Bale, & Casey, 2021). Drug and alcohol intake are also assessed, with alcohol dependency (with and without physical symptoms) or excessive use (greater than the maximum recommended number of units per week) documented.

Data were extracted for all assessed presentations over an eleven-year period (2010–2020) where the individual’s occupation was coded as a current or former nurse or midwife. These data were merged from two databases, the main monitoring database, which at the time of identifying data for the study included presentations in 2010–2017, and an additional database for which data for 2018–2020 were obtained through hand-searching of monitoring system data forms not yet entered into the main database. After merging of the databases, the data were cleaned.

### Data Analysis

Data were analyzed using SPSS version 22 for Windows. Descriptive analyses were conducted. Data are presented as frequencies and percentages. In a minority of cases, there were missing data due to incomplete information recorded during psychosocial assessment. Variables with missing data are highlighted within the results. Where individuals presented following multiple self-harm episodes during the study period, the first episode was used as the index.

### Ethical Approval

The Oxford Self-Harm Monitoring System has approval to collect data on self-harm for local monitoring and research purposes. The system is compliant with the General Data Protection Regulation (2018) and has approval under Section 251 of the National Health Service (NHS) Act (2006) to collect patient-identifiable information without explicit patient consent.

## Results

### Sample

Across the eleven-year study period (2010–2020), 81 current or former nurses (*N* = 74) and midwives (*N* = 7) presented to the hospital following 107 episodes of self-harm (Table 1). Most individuals (67, 82.7%) had one presentation during the study period. Some had repeat presentations (14, 17.3%), ranging from two to six episodes. The numbers of first presentations per year varied (median 8, 3–11 range), including a relatively small number in 2020 (*N* = 5), the first year of the COVID-19 pandemic.

**Table 1:** Characteristics of presenting nurses and midwives by occupation, sex, and marital status

Characteristic	Employed *N* = 47	Unemployed *N* = 18	Sick *N* = 5	Retired *N* = 8	In other employment *N* = 3	Total *N* = 81
Profession *N* (%)						
Nurse	46 (56.8%)	15 (18.5%)	4 (4.9%)	6 (7.4%)	3 (3.7%)	74 (91.4%)
Midwife	<3	3 (3.7%)	<3	<3	0	7 (8.6%)
Sex *N* (%)						
Female	44 (54.3%)	15 (18.5%)	5 (6.2%)	8 (9.9%)	3 (3.7%)	75 (92.6%)
Male	3 (3.7%)	3 (3.7%)	0	0	0	6 (7.4%)
Marital status *N* (%)						
Single	24 (29.6%)	7 (8.6%)	3 (3.7%)	<3	<3	38 (46.9%)
Married	13 (16.0%)	6 (7.4%)	<3	4 (4.9%)	<3	25 (30.9%)
Separated/divorced	10 (12.3%)	5 (6.2%)	<3	<3	0	17 (21.0%)
Widowed	0	0	0	<3	0	<3

Most of the individuals were female (75, 92.6%; see Table 1) and were of White ethnicity (75, 92.6%). Their ages ranged from 21 to 93 years, with a mean of 40.6 (*SD* 14.6), and median of 39.0. Most patients were working as current nurses or midwives (47, 58.0%) at the time of hospital presentation. However, a substantial proportion were currently unemployed (18, 22.2%). Others were on sick leave (5, 6.2%), working in another position (3, 3.7%), or retired (8, 9.9%).

### Self-Harm Methods

The majority of first self-harm presentations involved self-poisoning (58, 71.6%), with just over a fifth involving self-injury (18, 22.2%). A further small proportion involved both self-poisoning and self-injury in the same episode (5, 6.2%).

The group of drugs most frequently used for self-poisoning (including co-occurring self-poisoning and self-injury) were antidepressants (24/63, 38.1%), with fluoxetine, mirtazapine, and amitriptyline being the individual drugs most used. Pure paracetamol and paracetamol-containing drugs (23/63, 36.5%) were next most frequent, followed by opiates or other recreational drugs (13, 20.6%), minor tranquillizers and hypnotics (12, 19.0%), other “over-the-counter” or prescribed drugs, (10, 15.9%), and other analgesics (including NSAIDs; 9, 14.3%). Other substances used by small numbers of individuals were mood stabilizers, major tranquilizers, and noningestible poisons (including gas). For two patients (3.2%), the substance taken was unknown. Many patients used multiple types of drugs within a single episode (24/63, 38.1%). When restricting analyses to only nurses and midwives currently employed within the profession, the drugs used for self-poisoning were not different.

Of all episodes involving self-injury (*N* = 23), three (13.0%) included the use of multiple methods. Across all episodes of self-injury (including where multiple methods were used within a single episode), self-cutting of the wrist and/or forearm was the most common method (13/23, 56.5%). Several other methods of self-injury were also used, including cuts to other areas of the body (5/23, 21.7%). Attempted hanging and drowning were among other less common self-injury methods.

Of patients with multiple presentations (*N* = 14), half (7, 50.0%) used the same broad method of self-harm (self-poisoning or self-injury) in each self-harm episode, five (35.7%) changed methods across episodes, and two (14.3%) moved from using one method of harm to using self-poisoning and self-injury simultaneously.

Many patients had consumed alcohol within the six hours before self-harming (35/80, 43.8%). A smaller proportion reported using alcohol as part of the episode (19/75, 25.3%). Only one patient was known to be under the influence of recreational drugs at the time of self-harm.

### Psychiatric History

At first presentation during the study period, just over a quarter of patients were in receipt of outpatient or day patient psychiatric care (21/79, 26.6%). One individual was a psychiatric hospital inpatient. Nearly a quarter had received previous inpatient care (18/76, 23.7%), and a half outpatient care (38/75, 50.7%). Half of the patients were identified as having a psychiatric disorder (36/71, 50.7%), with a smaller proportion having a personality disorder (13/59, 22.0%).

#### Previous Self-Harm

Over two-thirds of patients had a known history of previous self-harm (54/78, 69.2%), including nearly half with hospital-presenting self-harm (37/76, 48.7%). Where information on recency of previous self-harm was available (*N* = 47), a quarter of patients had self-harmed within the last month (12/47, 25.5%).

#### Substance Misuse

Excessive consumption of alcohol was relatively common (18/79, 22.8%), with a few additional cases of alcohol dependency (8/79, 10.1%). Drug use was rare, with only five patients reporting use (6.2%). The most common substances were opiates, followed by cocaine, benzodiazepines, and cannabis, all of which were reported in less than five cases.

### Suicide Intent

Data regarding scores on the Suicide Intent Scale were available for 86.4% (*N* = 70) of first presentations. The median suicide intent score was 8.5 (range 0–28). The scores of a third of patients (23/70, 32.9%) indicated low suicide intent, another third (25/70, 35.7%) moderate intent, one fifth (14/70, 20.0%) high intent, and eight presentations (11.4%) were classified as having very high suicide intent.

### Problems Faced by Individuals

The most common problems nurses and midwives were facing at the time of self-harm were relationship difficulties with a partner, these being identified in nearly half of the patients (38, 46.9%). The next most common problem was related to psychiatric disorder (24, 29.6%). Approximately a quarter of the patients had problems related to employment (22, 27.2%), and a similar proportion had problems in their relationship with their families (20, 24.7%). No nurses or midwives identified having problems concerning sexual adjustment or because of being a carer (see Table 2). At the time of presentation, just over one-fifth of patients reported having experienced violence from others in the previous five years (15/72, 20.8%), and a small proportion had been violent toward others (6/74, 8.1%).

**Table 2:** Problems identified in nurses and midwives after first self-harm presentation

Problem	*N* (%)^a^
Relationship with partner	38 (46.9%)
Psychiatric disorder	24 (29.6%)
Employment	22 (27.2%)
Relationship with family	20 (24.7%)
Alcohol	19 (23.5%)
Physical health	16 (19.8%)
Finance	15 (18.5%)
Social isolation	13 (16.0%)
Bereavement	11 (13.6%)
Housing	8 (9.9%)
Childhood sexual abuse	7 (8.6%)
Drugs	7 (8.6%)
Eating disorder	7 (8.6%)
Relationship with friends	6 (7.4%)
Childhood emotional abuse or neglect	4 (4.9%)
Childhood physical abuse	4 (4.9%)
Chronic pain	4 (4.9%)
Legal	<3
Repetitive self-mutilation	<3
Bullying^b^	<3
Sexual adjustment	0
Caring duties^b^	0
*Note*. *N* = 81. ^a^Multiple problems were recorded for some patients. ^b^19.8% of data missing; measures were added from 2012 onward.	

A large proportion of patients reported physical health conditions (33/78, 42.3%), with most common conditions including musculoskeletal (7/78, 9.0%), respiratory (6/78, 7.7%), and gynecological problems (5/78, 6.4%). Multiple health conditions were reported by 10 patients (10/78, 12.8%).

### Aftercare Offered

Following their first presentation, approximately half of the patients were offered outpatient care (41, 50.6%) and nine (11.1%) were admitted to psychiatric inpatient care. A small proportion were referred to the Improving Access to Psychological Therapies (IAPT) national psychological counseling program (8, 9.9%), at times alongside other aftercare. Over a third of patients were referred to their general practitioners, (30, 37.0%); for some, this was alongside other aftercare offers (12 /30, 40.0%). Small proportions were referred to alcohol services (6, 7.4%) or social services.

## Discussion

We examined characteristics of nurses and midwives who presented to hospital following self-harm over the eleven-year period. Most individuals were female (92.6%). While we were unable to estimate the relative rates of self-harm by sex, it is recognized that risk of suicide is elevated in female nurses in the United Kingdom (Windsor-Shellard & Gunnell, 2019). The final year of the study period saw the beginning of COVID-19. This may have been a reason for the relatively small number of self-harm presentations in 2020, as hospital presentations for self-harm in general were reduced during the initial phase of the pandemic (e.g., Hawton, Casey, et al., 2021).

The majority of self-harm episodes involved self-poisoning. This is consistent with research on suicide methods of female nurses (e.g., Davidson et al., 2020; NCISH, 2020). The common use of self-poisoning contrasts with a self-report study of self-harm among nurses, where cutting and hitting were most frequent (Cheung & Yip, 2016). This difference may be a result of the previous study including self-reported self-harm, not just hospital presentations. Self-injury including cutting is far more common in self-harm episodes in the community, whereas self-poisoning is more frequent in individuals presenting to hospitals (Geulayov et al., 2018; McManus et al., 2019).

As in the current study, antidepressants, paracetamol (acetaminophen), and opiates or other recreational drugs have been shown to be commonly used for self-poisoning among nurses (e.g., Davidson et al., 2020). In the present sample, the frequent use of antidepressants may reflect the fact that many of the individuals were experiencing psychiatric disorders, especially depression, at the time of self-harm, as found in nurses who died by suicide (Hawton et al., 2002). The use of opiates (recreational and nonrecreational) may be linked to the prevalence of musculoskeletal or pain-related conditions, for which nurses are at high risk (Davis & Kotowski. 2015).

The present study contributes to the discussion of whether self-poisoning among nurses is related to access to medication in the workplace and/or knowledge regarding lethality. Like other studies examining suicide in nurses (Davidson et al., 2020), prescribed or over-the-counter medications (other than recreational opiates) were most frequently used, suggesting that workplace access is not often a contributor, unlike suicidal behavior in other health-related professions, such as anesthetists (e.g., Plunkett et al., 2021).

Alcohol was frequently used prior to and as part of the self-harm acts, and alcohol misuse was relatively common. Postulated reasons for excessive alcohol use among nurses include work stress (Foli et al., 2020) and use as a sleep aid (Dorrian et al., 2011). In the assessment of nursing staff who have self-harmed or are thought to be at risk, alcohol use should be considered given research demonstrating high levels of problematic use among nurses who have died by suicide (e.g., Hawton et al., 2002; NCISH, 2020), and use of alcohol as part of suicide acts (e.g., Davidson et al., 2020).

The proportion of nurses and midwives with a history of self-harm was considerable, with over two-thirds (69.2%) having at least one previous episode, a higher proportion than middle-aged patients (men 53.5%, women 57.6%) in the general population presenting to hospitals in England (Clements et al., 2019). Previous episodes were often relatively recent (most not involving hospital presentation). Furthermore, repeat episodes leading to further hospital presentation during the study period were quite common. This is concerning given the association and commonality of prior self-harm and later suicide among nurses and midwives (Hawton et al., 2002; NCISH, 2020), the known increased risk of suicide associated with repetition of self-harm (Geulayov et al., 2019), and the substantial proportion of self-harm presentations in the present study with high or very high suicidal intent. Median suicide intent scores in the current sample (score 8.5) were similar to females presenting to the same hospital in previous studies of self-harm (scores of 8 in both; Harriss et al., 2005; Haw et al., 2015).

Most of the nurses and midwives had previous contact with psychiatric services, and a large proportion had psychiatric disorders. Comparing the nurses with the general population of patients presenting to the same hospital following self-harm, the nurses had a higher prevalence of psychiatric disorders (50.7% vs. 29.4%), personality disorders (22.0% vs. 8.7%), and previous inpatient psychiatric service contact (23.7% vs. 16.4%) (Haw et al., 2015; Haw & Hawton, 2008). While we did not have detailed information about specific conditions, anxiety, depression, and panic disorder have been linked to suicidal behaviors among nurses (e.g., Stelnicki et al., 2020). This highlights the need for appropriate support and treatment for nurses experiencing mental health conditions.

Relationship difficulties were the most common problem identified by nurses and midwives. This correlates with other studies of self-harm (Cheung & Yip, 2016) and suicide among nurses (e.g., Hawton et al., 2002). Employment-related issues were also highly prevalent among nurses and midwives (27.2%), higher than midlife men (21.7%) and women (13.5%) in general presenting to hospitals in England following self-harm (Clements et al., 2019). Specific problems among nurses may include job loss, work-related stress, and stigma related to seeking support (Davidson et al., 2021; Wolf et al., 2020). Physical health problems and comorbidity were common within the sample. Reasons for this, particularly regarding musculoskeletal conditions, may include high physical demands of nursing, for example, frequent lifting and long periods spent standing. Health problems including chronic pain have been found to contribute to suicide in nurses (e.g., Davidson et al., 2021).

### Strengths and Limitations

The study findings are based on detailed and systematic clinical assessments, with the method of data collection being consistent across the study period. However, detailed information was only available on nurses who had a psychosocial assessment. Due to the relatively small number of nurses and midwives presenting, only descriptive analyses were appropriate. Therefore, this limits generalizability of the results. Nurses and midwives who self-harmed in the community and did not present to hospital will not have been included in the study. Their characteristics, including the method used for self-harm, are likely to differ from those of nurses and midwives presenting to hospital following self-harm (Cheung & Yip, 2016).

The information obtained for this study was largely based on a single psychosocial assessment. Data collected from the assessment may have limitations, including possible inaccuracies in recording, and misattribution by clinical staff of potential contributory factors. Also, we only had information on aftercare offered, not received. The results of the study are limited by being from a single center in a relatively affluent area (Geulayov et al., 2019). Participants of the study were largely of White ethnicity (92.6%), a higher proportion than Oxford’s population (78%; Oxford City Council, 2011) and the NHS nonmedical workforce, in which nursing is included (80.3%; NHS Workforce, 2021).

### Implications for Research and Policy

Further research should explore the nature of and interaction between problems that nurses may identify as precedents to self-harm, for which qualitative approaches may be appropriate. Although case–control studies have been conducted examining characteristics of nurses who have died by suicide (e.g., Hawton et al., 2002), to the authors’ knowledge, characteristics of those self-harming have not been examined in this manner. A case–control study comparing characteristics of self-harm among nursing professionals with the general population or a reference occupation could clarify whether any factors differ and thus may be particularly important for targeted interventions for self-harm and suicide prevention for nurses. A longitudinal study in which nurses and midwives who self-harm are followed up to assess aftercare received and its outcome would provide further understanding of the significance of self-harm in this population.

The findings from the present study have highlighted factors that may inform primary and secondary prevention initiatives to target problems nurses and midwives might face, in turn contributing to the prevention of self-harm and suicide. Primary intervention initiatives should include education regarding stress management, injury prevention, and safe levels of alcohol use. Secondary intervention initiatives could include support for nurses and midwives with harmful levels of alcohol use, ensuring continuous learning opportunities regarding moving and handling, tailored health promotion resources to increase awareness and reduce stigma surrounding mental health conditions, flexible and accessible workplace counseling, and robust clinical supervision arrangements.

## Notes

**Conflict of Interest:** KH declares grants from the National Institute for Health Research and the Department of Health and Social Care. He is a member of the National Suicide Prevention Strategy for England Advisory Group and is a National Institute for Health Research (NIHR) Senior Investigator (Emeritus). All other authors declare no competing interests. The views expressed are those of the authors and not necessarily those of the NHS, NHSE, NIHR, or the Department of Health and Social Care.

**Publication Ethics:** The Oxford Self-Harm Monitoring System has approval to collect data on self-harm for local monitoring and research purposes. The system is compliant with the General Data Protection Regulation (2018) and has approval under Section 251 of the National Health Service (NHS) Act (2006) to collect patient-identifiable information without explicit patient consent.

**Authorship:** Samantha Groves, study conception and design, data analysis, interpretation of the results, original draft writing, critical review and report editing. Karen Lascelles, study conception and design, interpretation of the results, critical review and report editing. Liz Bale, acquisition of data, critical review and report editing. Fiona Brand, acquisition of data, critical review and report editing. Deborah Casey, acquisition of data, data analysis, critical review and report editing. Keith Hawton, study conception and design, acquisition of data, interpretation of the results, critical review and report editing. All authors approved the final report and are accountable for all aspects of this work.
